# Circular RNA as a Novel Regulator and Promising Biomarker in Polycystic Ovary Syndrome

**DOI:** 10.3390/biom13071101

**Published:** 2023-07-11

**Authors:** Tianrui Jing, Yifan Wu, Anran Wan, Chengmin Ge, Zi-Jiang Chen, Yanzhi Du

**Affiliations:** 1 Center for Reproductive Medicine, Ren Ji Hospital, School of Medicine, Shanghai Jiao Tong University, Shanghai 200135, China; tianrui_jing@163.com (T.J.); med-wyf@sjtu.edu.cn (Y.W.); wananran@renji.com (A.W.); gechengmin@renji.com (C.G.); chenzijiang@hotmail.com (Z.-J.C.); 2 Shanghai Key Laboratory for Assisted Reproduction and Reproductive Genetics, Shanghai 200135, China; 3 Center for Reproductive Medicine, Shandong University, Jinan 250012, China; 4 National Research Center for Assisted Reproductive Technology and Reproductive Genetics, Shandong University, Jinan 250012, China; 5 Key Laboratory of Reproductive Endocrinology of Ministry of Education, Shandong University, Jinan 250012, China; 6 Shandong Key Laboratory of Reproductive Medicine, Shandong Provincial Hospital Affiliated to Shandong First Medical University, Jinan 250012, China; 7 NMU-SD Suzhou Collaborative Innovation Center for Reproductive Medicine, Suzhou 215000, China

**Keywords:** PCOS, circRNAs, biological functions, biomarkers, therapeutic targets

## Abstract

Polycystic ovary syndrome (PCOS) is a prevalent metabolic and reproductive disorder that causes low fertility in females. Despite its detrimental effects on women’s health, care for PCOS has been impeded by its undefined pathogenesis. Thus, there is an urgent need to explore novel biomarkers and therapeutic targets for the diagnosis and treatment of PCOS. Circular RNAs (circRNAs) are a class of noncoding RNAs with covalently closed cyclic structures, present in high abundance, and show development-stage specific expression patterns. Recent studies have demonstrated that circRNAs participate in PCOS progression by modulating various biological functions, including cell proliferation, apoptosis, and steroidogenesis. In addition, circRNAs are widely present in the follicular fluid of women with PCOS, indicating their potential as diagnostic biomarkers and therapeutic targets for PCOS. This review provides the current knowledge of circRNAs in PCOS, including their regulatory functions and molecular mechanisms, and explores their potential as diagnostic biomarkers and therapeutic targets.

## 1. Introduction

Polycystic ovary syndrome (PCOS) is the most common endocrinologic disease, affecting 6–20% of reproductive-aged women [1]. It is a heterogeneous disease showing variable phenotypes and characterized by hyperandrogenism, polycystic ovarian morphology, and menstrual irregularity [2]. PCOS is also associated with metabolic disorders, including insulin resistance (IR), obesity, hyperlipidemia, type 2 diabetes mellitus, and cardiovascular disease [2]. There are pregnancy-related complication risks associated with PCOS, such as preterm birth, antepartum hemorrhage, gestational diabetes, and pregnancy-induced hypertension [3]. The etiology and pathogenesis of PCOS remain unclear, which poses challenges in diagnosis and treatment. Therefore, it is crucial to identify the underlying mechanisms of PCOS, explore its clinical biomarkers, and develop more effective treatment strategies.

The initiation and progression of PCOS are complex and associated with epigenetic modifications and genetic factors [4,5]. Noncoding RNAs (ncRNAs), key members of the epigenetic regulation network, have been reported to contribute to the development of PCOS, thus opening up new prospects for PCOS diagnosis, prognosis, and treatment [6]. Circular RNAs (circRNAs) are a new class of endogenous ncRNAs with a covalently closed structure, mainly synthesized by the transcription of protein-coding genes with RNA polymerase II (Pol II), and contain components of exons and/or introns from parental genes [7]. Unlike linear RNA, circRNAs lack 5′ caps and 3′ polyA tails, which make them more stable. They are dynamically expressed in a specific manner [8] and regulate various biological functions, including cell proliferation, apoptosis, differentiation, and metabolism [9,10]. CircRNAs have gradually demonstrated the potential to modulate target genes’ function and expression, which contribute to different diseases, including pregnancy-related diseases, metabolic disorders, cardiovascular disorders, cancer, and neurological disorders [11,12,13,14,15]. Therefore, circRNAs have been proposed as promising disease biomarkers.

Concerning their role in PCOS, circRNAs have been identified in ovaries [16,17] and proven to have development stage-specific expression patterns during follicular development [18,19]. CircRNAs have also shown differential expression in women with and without PCOS [20,21]. Accumulative evidence has demonstrated that circRNAs play an essential regulatory role in the progression of PCOS by means of diverse mechanisms [22,23,24]. Therefore, circRNAs may become dependable diagnostic and attractive therapeutic targets for PCOS. Nevertheless, evidence regarding the exact relationship between circRNAs and PCOS is still preliminary, and the potential functions of circRNAs in the diagnosis and therapy of PCOS have not yet been clarified.

In this review, we aimed to provide an overview of current knowledge on the relationships between circRNAs and PCOS, illustrating their diagnostic and therapeutic potentials.

## 2. The Biology of Circular RNAs

### 2.1. The Biogenesis of Circular RNAs

**Figure 1:**
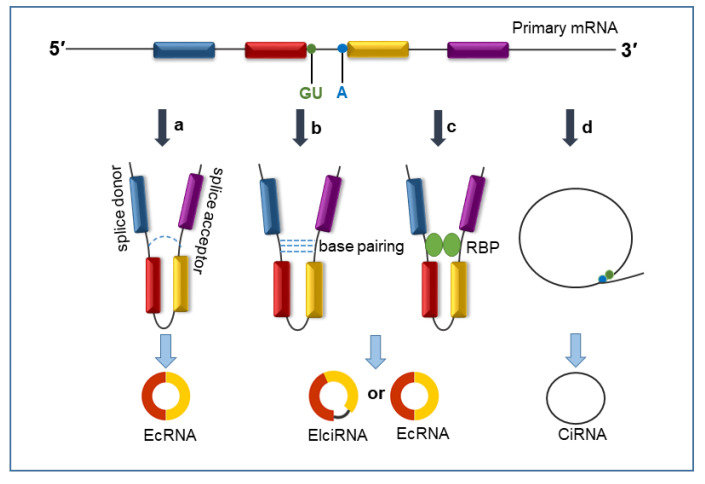
Biogenesis of circRNAs. (**a**) Lariat-driven circularization: the model requires covalently binding between the splice donor and splice acceptor, forming an exon-containing lariat. The spliceosome removes the introns to generate an exonic circRNA (EcRNA). (**b**) Intron pairing-driven circularization: intronic complementary base-pairing brings two adjacent exons close together and generates a circular structure. The introns are removed or retained to form an EcRNA or an exon-intron circRNA (EIciRNA). (**c**) RNA binding protein (RBP)-drived circularization: the interaction between two RBPs can interact with two flanking introns together and form a circular structure. The introns are removed or retained to form an EcRNA or EIciRNA. (**d**) Intron cyclization: the GU-rich element and C-rich element bind together to form a circular structure. The exons and introns in this area are removed by spliceosome to form a circular intronic RNA (ciRNA).

CircRNAs are primarily generated from the transcription of protein-coding genes with Pol II; unlike linear RNAs, they are not produced by the canonical mode of pre-mRNA splicing [25]. CircRNAs are circularized by unique back-splicing, in which the 3′-ends ligate to the 5′-ends, generating a covalently closed cyclic structure with a back-splicing junction site [7]. According to their different origins, circRNAs can be divided into three categories: exonic circRNAs (EcRNAs) that come from exons; intronic circRNAs (ciRNAs) that only contain introns; and exon–intron circRNAs (EIciRNAs) that are circularized with both exons and introns. EIciRNAs and ciRNAs are located in the nucleus [26,27], while most EcRNAs are mainly exported to the cytoplasm after their biogenesis [28]. Two different models of circRNA biogenesis have been proposed and validated, which are called “lariat-driven circularization” (Figure 1a) and “intron pairing-driven circularization” [29] (Figure 1b). In addition, recent studies have indicated that RNA molecules with RNA-binding proteins (RBPs) are involved in EcRNA and EIciRNA biogenesis [30] (Figure 1c). The biogenesis of ciRNAs is based on the 7nt GU-rich element near the 5′ splice site and the 11nt C-rich element close to the branchpoint site. The GU-rich element and the C-rich element bind together to form a circular structure. Then, the exons and introns in this area are removed by spliceosome to form a ciRNA [31] (Figure 1d).

### 2.2. The Function of Circular RNAs

**Figure 2:**
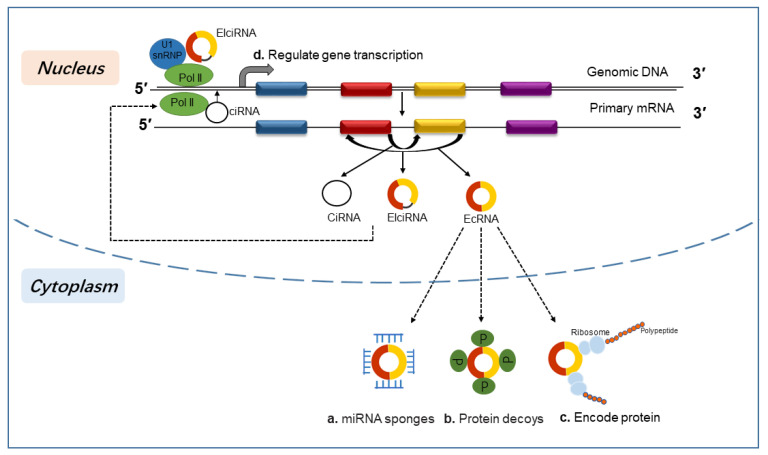
Mechanisms of circRNA function. (**a**) CircRNAs can act as miRNA sponges. circRNAs can competitively bind miRNAs and subsequently suppress the function of miRNAs on target genes. (**b**) CircRNAs can interact with RNA-binding proteins (RBPs) that serve as protein baits, thus arresting the function of proteins. (**c**) CircRNAs can encode polypeptides in a cap-independent manner. (**d**) EIciRNAs can interact with U1 snRNP and RNA polymerase II (Pol II) to promote their parental gene transcription. CiRNAs can directly interact with Pol II to induce their parental gene transcription.

CircRNAs have multiple biological functions exerted via various molecular mechanisms. Many studies have proven that circRNAs can exert function by acting as microRNA (miRNA) sponges [7] (Figure 2a). CircRNAs have plenty of RNA response elements that allow them to competitively combine with miRNAs, acting as intracellular competitive endogenous RNAs (ceRNAs), leading to the inhibition of miRNA function, and then upregulating the target gene of miRNAs [32]. Although “miRNA sponge” has been regarded as a classical model for circRNA function, some studies have proven that most circRNAs do not exhibit the sponge effect, and physiological ceRNA expression changes have no impact on highly expressed miRNAs [33,34]. In addition to the function of miRNA sponges, circRNAs can also interact with RNA-binding proteins to facilitate protein translation of target mRNAs [35] (Figure 2b). CircRNAs can interact with mRNAs as well. They have translation start sites that make them act as mRNA traps to sequester the translation start sites and thereby regulate protein translation of mRNA [36]. Although circRNAs are regarded as ncRNAs, convincing evidence has revealed their translational function [37] (Figure 2c). Some circRNAs have stable open reading frames and binding sites of ribosomes, suggesting their translational potential [38]. Due to the absence of a 5′ cap and 3′ polyA tail, circRNAs can only be translated by cap-independent translation [39]. Internal ribosome entry site (IRES)-mediated translation is one of the mechanisms of circRNA translation initiation [40]. Another important cap-independent translation mechanism is N6-methyladenosines (m6A) in the 5′ UTR, and a single m6A residue is sufficient to initiate circRNA translation [41]. m6A in the 5′ UTR can directly interact with eukaryotic initiation factor 3, which is sufficient to recruit the 43S preinitiation complex, and initiate translation in a cap-independent manner [42]. Additionally, bioinformatics tools have identified the universal existence of circRNAs with coding potential [43,44] and provide new insights into circRNA-coding proteins. Although the majority of circRNAs are transported to cytoplasm, circRNAs that contain introns are restricted in the nucleus and regulate parental gene transcription via distinctive mechanisms (Figure 2d). EIciRNAs and ciRNAs both regulate parental gene transcription in cis-regulatory effect. EIciRNAs enhance Pol II transcriptional activity of their parental gene by interacting with U1 small nuclear ribonucleoprotein [26]. CiRNAs gather in the transcriptional sites to interact with Pol II machinery and then promote their host gene expression [27].

## 3. Circular RNAs Regulate the Development of PCOS

With the rapid development of detecting technology and computational analysis, a large number of circRNAs have been identified in different diseases [45]. In general, the procedure of detecting circRNAs in PCOS can be briefly summarized as follows: firstly, using whole-genome sequencing techniques such as high-throughput microarrays or high-throughput RNA-seq to sequence the genome of circRNAs in PCOS-related tissues or cells, then validating the back-splicing junction site and expression level of circRNAs using sanger sequencing and reverse transcription-polymerase chain reaction (RT-PCR), respectively. Finally, Gene Ontology (GO) analysis, Kyoto Encyclopedia of Genes and Genomes (KEGG) pathway analysis, or other methods are used to predict the biological functions and mechanisms of these circRNAs in PCOS. Multiple dysregulated circRNAs were significantly differentially expressed in granulosa cells (GCs), cumulus cells (CCs), and follicular fluid (FF) in PCOS patients compared with non-PCOS population (Table 1). Recent studies have suggested that circRNAs play an essential role in the development of PCOS and can potentially function as biomarkers for the diagnosis and treatment of PCOS.

### 3.1. Circular RNAs and Ovarian Dysfunction in PCOS

PCOS patients classically show GC dysfunction, abnormal folliculogenesis, and ovulation disorder [51]. Emerging evidence suggests that circRNAs regulate GC function and folliculogenesis in physiology and pathology. CircRNAs have been found to be expressed in a stage-specific manner of GCs during follicular development and act as miRNA sponges to regulate multiple reproductive pathways involved in GnRH signaling, oocyte meiosis, and progesterone-mediated oocyte maturation [19]. Furthermore, circRNAs have been suggested to be involved in oocyte genesis and quality. For example, the expression of circ_103827 and circ_104816 in GCs were positively associated with maternal age and negatively correlated with serum anti-Mullerian hormone (AMH) level, number of sinus follicles, and oocyte quality [52]. In animal models, circARMC4 knockdown significantly impaired chromosome alignment during porcine oocyte meiotic maturation, and thus damaged porcine embryo development [53]. Thus, these circRNAs may regulate ovarian function and be potential indicators to reflect damage to the follicular microenvironment.

**Figure 3:**
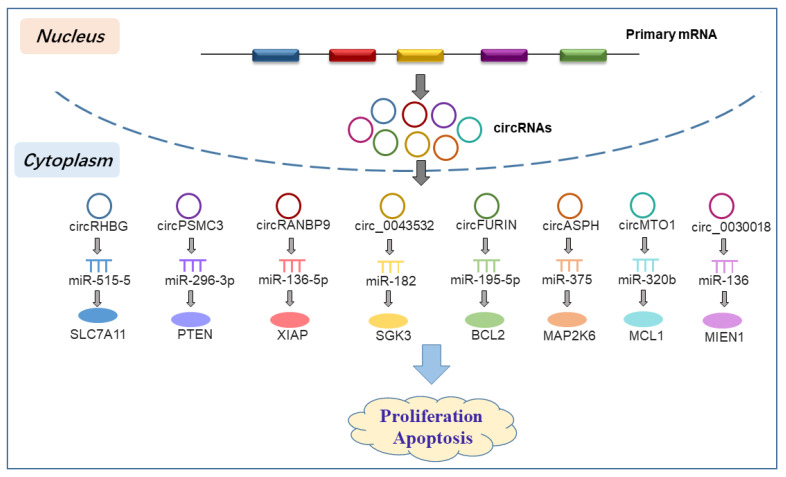
CircRNAs in miRNA-associated gene regulatory networks that regulate granulosa cell proliferation and apoptosis to participate in the progression of PCOS.

The balance between proliferation and apoptosis of GCs is the fundamental cause of follicular development and atresia [54]. Altered expression of circRNAs can modulate the balance by serving as miRNA sponges in PCOS (Figure 3). A vast array of circRNAs have been explored to determine their functions in the proliferation of GCs, as well as the cell cycle and apoptosis in PCOS. For instance, a study observed a significant decrease in circPSMC3 levels in ovarian tissue samples from PCOS patients compared with non-PCOS individuals. In vitro functional experiments indicated that overexpressed circPSMC3 could inhibit cell proliferation, suppress cell cycle, and promote apoptosis in human-like granular tumor cell lines (COV434 and SVOG). Further investigations confirmed that circPSMC3 exerted its function by sponging miR-296-3p to regulate PTEN expression. CircMTO1 significantly influenced proliferation and apoptosis rate of GCs through miR-320b, which directly targeted MCL1 in PCOS [55]. Similarly, the expression of circ_RANBP9 was closely associated with GC proliferation and apoptosis; subsequent studies revealed that circ_RANBP9 functioned via the miR-136-5p/XIAP axis in PCOS [56]. Another study demonstrated that deficiency of circ_0043532 markedly inhibited cell proliferation and cell cycle process and promoted cell apoptosis in PCOS by targeting the miR-182/SGK3 axis [57]. Furthermore, the same circRNA can regulate GC function by a different miRNA-mRNA axis in PCOS. For example, circ-FURIN, derived from exon 16 of the FURIN gene on chromosome 15, was significantly upregulated in PCOS. It inhibited cell proliferation, induced cell cycle arrest, and facilitated apoptosis rate of human ovarian granulosa-like cells (KNG) in PCOS via the miR-423-5p/MTM1 axis [22]. Another study identified that circ-FURIN modulated BCL2 expression by acting as a ceRNA for miR-195-5p binding in PCOS progression [58]. These researches pointed out the importance of circRNAs in regulating the proliferation, cell cycle, and apoptosis of GCs, as well as folliculogenesis, and suggested their potential as targets for assessing ovulation in PCOS.

### 3.2. Circular RNAs and Steroidogenesis in PCOS

Androgens, including testosterone, dihydrotestosterone, and androstenedione, exert a vital function on ovulation by facilitating follicular development and maturation [59]. However, excess androgen causes ovulatory and metabolic dysfunction in PCOS [60]. Hyperandrogenism drives IR and visceral adiposity, which in turn increases ovarian androgen production [51]. Androgen exerts its actions by interacting with the androgen receptor (AR) in different target tissues. The increased expression of AR has been detected in PCOS patients [61]. A bulk of evidence indicates that circRNAs have functional interactions with AR and regulate AR expression in specific tissues [62], but further exploration is needed to understand the effects of circRNAs on AR in PCOS. The function of circRNAs on steroidogenesis from ovarian cells has been explored. One circRNA, circLDLR, is generated from its parental gene low-density lipoprotein receptor (LDLR), significantly depleted in FF exosomes of PCOS patients. In a medium containing recipient KGN cells, estradiol secretion was significantly inhibited by treatment with silencing circLDLR exosomes. In contrast, by treatment with overexpressed circLDLR exosomes, estradiol secretion was markedly elevated [50].

Steroid hormones are synthesized from cholesterol regulated by a series of genes involved in the regulation of androgen metabolism and lipid transport [63]. Key genes, such as CYP19, CYP11A, CYP19A1, and 3β-HSD, are involved in steroid hormone synthesis and estrogen production regulated by the CYP19A1 gene. For example, depletion of circLDLR downregulated CYP19A1 to inhibit estradiol production by targeting miR-1294 in PCOS [50]. Another circRNA, circDDX10, was found to regulate estradiol synthesis in GCs, with silencing circDDX10 inhibiting estradiol synthesis and causing a simultaneous decrease in CYP11A1 and CYP19A1 expressions. In contrast, overexpression of circDDX10 increased CYP19A1 and HSD17B1 expression, resulting in increased estradiol synthesis [64]. Therefore, further exploration of the connection between circRNAs and steroidogenesis will contribute to the diagnosis and treatment of PCOS.

### 3.3. Circular RNAs and Follicular Fluid in PCOS

Follicular fluid (FF) serves as an essential microenvironment for oocyte development and maturation [65]. Its biochemical composition contains secretory products and metabolites of oocytes and various hormones, including androgen, estrogen, luteinizing hormone (LH), follicle-stimulating hormone, and AMH [66]. Therefore, the components of FF can reflect the quality and physiological status of the follicle.

Recent studies have shown that a large number of circRNAs were differentially expressed in the FF of women with PCOS compared with normal control women. A study detected 16,771 circRNAs, of which 167 were upregulated and 245 downregulated in the FF of PCOS patients. The parental genes of circRNAs can target signaling pathways, including bacterial infection, associated chronic inflammation, and oxidative stress [20]. Xin Huang et al. identified 16 differentially expressed circRNAs, of which 5 were upregulated and 11 were downregulated in the FF of PCOS patients compared with non-PCOS control. GO and KEGG pathway analysis indicated that the parental genes of differentially expressed circRNAs were enriched in PCOS-related pathways, including ovarian aldosterone synthesis and secretion, steroidogenesis, and Jak-STAT signaling. Among the differentially expressed circRNAs, the depletion of circLDLR was further verified by RT-PCR using the FF of oocytes collected from another 25 PCOS patients and 25 non-PCOS patients. Furthermore, the inhibition of circLDLR in FF exosomes increased miR-1294 expression, reduced CYP19A1 expression in recipient cells, and inhibited their estradiol production [50]. Therefore, examining circRNAs present in the FF of PCOS patients may provide a novel biomarker for the diagnosis of PCOS.

## 4. The Possible Roles of Circular RNAs in PCOS

### 4.1. Circular RNAs and Insulin Resistance

IR, a common metabolic feature in PCOS, occurs in up to 70% of PCOS patients. It is an important aggravating factor in PCOS pathogenesis and contributes to an increase risk of metabolic syndrome and metabolism-related disorders [67]. Hyperinsulinism acts on theca cells (TCs) to stimulate steroidogenesis and promote ovarian androgen secretion [67], while also provoking LH excess and potentiating LH effect on TCs and GCs, further worsening hyperandrogenism [68].

There is sufficient evidence supporting that circRNAs regulate insulin secretion and pancreatic β-cell function [12]. A large number of circRNAs have been identified in human islets, of which circCIRBP has been proven to regulate the insulin secretory index negatively [69]. CDR1as was enriched in islet β-cells and sponged miR-7, subsequently increasing the expression level of miR-7′s downstream target PAX6, leading to a significant increase in islet function [70]. Indeed, PAX6 was markedly upregulated, induced by high-level insulin in the endometrial tissues of PCOS patients, and promoted insulin-driven endometrial epithelial proliferation [71]. Another study found that circHIPK3 was the most abundant circRNA in pancreatic islets, and its depletion inhibited β-cell proliferation and insulin secretion [72]. Importantly, circHIPK3 was also presented in human ovarian tissues, and its silencing promoted proliferation, migration, and invasion, whereas it inhibited the apoptosis of ovarian cancer cells [73]. Additionally, circRNAs can regulate insulin signaling in target tissues. For example, circHIPK3 was found to promote IR by upregulating the expression of FOXO1 [74]. FOXO1 is a feedback regulator of the insulin signaling pathway that regulates hyperglycemia, hyperinsulinemia, hepatosteatosis, and IR [75,76]. Moreover, FOXO1 was significantly increased in CCs of PCOS women and involved in the pathogenesis of PCOS through various signaling pathways [77]. These findings indicated a strong association between circRNAs and IR, which may provide potential therapeutic targets for the management of IR in PCOS.

### 4.2. Circular RNAs and Lipid Disorders

Among PCOS patients, approximately 70% exhibit an abnormal lipid profile that manifests as high triglyceride, increased low-density lipoprotein cholesterol, and decreased high-density lipoprotein cholesterol levels [78,79]. These lipid disorders worsen IR, stimulate testosterone production, and suppress gonadotropin secretion, thereby contributing to PCOS pathogenesis [1]. Furthermore, abnormal lipid profiles increase the risk of cardiovascular morbidity in women with PCOS [80].

Numerous studies have established that circRNAs are involved in lipid metabolism and adipogenesis [81]. A study by Sun et al. revealed that a total of 4080 circRNAs were differentially expressed between human visceral preadipocytes and adipocytes. The parental genes of these circRNAs were enriched in fatty acid degradation and biosynthesis pathways [82]. Analysis of HepG2-based hepatocellular steatosis indicated that circRNA_0046367 and circRNA_0046366 abolished the suppressive effect of miR-34a on PPARα [83,84], a transcription factor that activates lipid metabolism-related genes, thus ameliorating hepatocellular steatosis [83]. Conversely, depletion of circScd1, which is upregulated in nonalcoholic fatty liver disease tissue, increased the degree of hepatocellular lipidosis and inhibited the levels of signal transducer and activator of transcription 5 (STAT5) and Janus kinase2 (JAK2) [85], which are essential for lipid-related metabolism [86,87]. Furthermore, several studies indicated that circRNAs could regulate adipogenesis [12]. For example, hsa_circ_0136134 was upregulated in human adipocytes compared with preadipocytes induced from visceral fat tissues. The parental gene of hsa_circ_0136134 is lipoprotein lipase (LPL), a crucial enzyme of adipocyte tissue triglyceride metabolism, implying that hsa_circ_0136134 may modulate the activity of LPL to take part in adipogenesis. In a study by Zhu et al., circH19 depletion significantly increased lipid droplet biogenesis and adipogenic gene expression, terminally leading to the adipogenic differentiation of adipose-derived stem cells [88]. These results imply that circH19 regulates the switch of adipose-derived stem cells from clonal expansion to terminal differentiation and may be a potential therapeutic target for dysregulated adipogenesis.

Although several circRNAs have been associated with lipid metabolism and adipogenesis in obesity, there is still a severe lack of evidence for a causative effect on obesity in PCOS patients. Further investigation is needed to determine the causal effect of circRNAs on obesity and their contribution to PCOS.

### 4.3. Circular RNAs and Fertility

There is sufficient evidence supporting that several circRNAs are involved in the regulation of the fertilization process and embryogenesis [11]. Thousands of circRNAs have been found in many mammalian embryos, such as those of mice, rabbits, and humans. It was uncovered that a total of 10,032 circRNAs were expressed in early human embryos using single-cell universal polyA-independent RNA sequencing technology. GO analysis indicated that the circRNA parental genes were enriched in chromosome and organelle organization, cell cycle process, and mitotic cell cycle. Further analysis suggested that these circRNAs were highly conserved in early embryos of different species, indicating that various animal models were suitable for research on circRNAs function in embryonic development [89]. Similarly, in a mouse model, 2891 circRNAs were detected in early embryos and their expression levels changed in different development stages. The average number of circRNAs presented dynamic change with development stages; they decreased from zygote stage to four-cell stage followed by an increase in morula, and then significantly decreased in blastocysts. The parental genes of these stage-specific circRNAs were enriched in cell division, cell cycle, DNA damage stimulus and repair, and chromosome organization [90]. In a rabbit model study, 744 circRNAs were differentially expressed between three stages of embryo development, and the expression of circRNA_07129, circRNA_15209, and circRNA_12526 progressively increased [91], with their targeted co-expressed mRNAs, WNT3, TBX1, and FGFR2, being involved in the regulation of embryonic development [92,93,94]. Therefore, circRNAs may regulate the development of tissues and organs in early animal embryos.

Moreover, implantation is a major factor for fertility and successful establishment of pregnancy, and circRNAs have been shown to regulate this process. A study showed that circRNAs_39503 and circRNAs_39505 were upregulated, whereas circRNAs_44122 and circRNAs_44123 were downregulated at implantation sites compared with interimplantation sites in day 5 pregnant mice [95]. The findings may lay a theoretical foundation for future exploration of circRNAs in embryo implantation.

### 4.4. Circular RNAs and Circadian Rhythm

A study suggested that the genome-wide rhythmic expression pattern of ovarian granulosa cells is disrupted in women with PCOS [96]. Our group also found that the classical dehydroepiandrosterone-induced PCOS rat model had peripheral biological clock disorders [97]. When abnormal light signals induce desynchronization of the body’s intrinsic rhythm and the external environment, rats develop PCOS-like reproductive and endocrine phenotypes, as well as alterations in the composition and diversity of the intestinal flora [98,99]. These findings demonstrate that biological clock rhythm disorders are highly involved in the development of PCOS, but the underlying mechanisms have not been studied in detail.

Various hormones in the body, such as cortisol and melatonin, exhibit circadian rhythmic changes, and this fluctuation plays an important role in maintaining physiological reproductive functions [100]. Pregnant women who perform shift work or with circadian rhythm disorders have abnormal melatonin secretion, which can lead to an increased risk of complications, such as preterm labor and preeclampsia. Melatonin or melatonin receptor 1A has been widely reported to improve PCOS by modulating AR and key metabolic enzymes [101]. An in vitro study showed that circ-ERC2 regulates rhythmic melatonin production in rat pineal glands via the miR-125a-5p/MAT2a axis [102]. In addition, animal experiments have shown that continuous light exposure leads to altered circRNA expression profiles in rat ovaries. Further bioinformatics analysis has shown that circRNAs and miRNAs may lead to ovarian dysfunction by regulating ovarian function-related signaling pathways, such as androgen receptor regulation and the GnRH pathway, under continuous light exposure [103]. Collectively, we hypothesize that circRNAs may play a role in rhythm disorder-induced PCOS development.

## 5. Circular RNAs as Potential Biomarkers of PCOS

PCOS is the main cause of female infertility, and therefore early diagnosis and proper therapeutic intervention are critical for the proper management of PCOS. Accumulating evidence has suggested that circRNAs could be used as excellent indicators of diagnosis and therapeutic targets for PCOS (Table 2).

An appropriate biomarker should possess stability, universality, specificity, and easy detectability. CircRNAs exhibit these properties, making them potential biomarkers for human diseases [106]. Firstly, circRNAs have high stability compared to linear RNAs [7], owing to their covalently closed loop structure that resists degradation by exonuclease RNase R [107]. Additionally, their half-life in plasma is longer than mRNA, surpassing 48 h [36]. Furthermore, circRNAs are evolutionally conserved in different species [108], which makes them suitable for clinical applications. Secondly, circRNAs are found to be universal molecules in human tissues and cells, and in some situations, circRNAs are more abundant than their linear mRNA [109]. In particular, a large number of circRNAs have been identified in reproduction-related tissues and cells [11]. According to current knowledge, circRNAs present tissue-specific and development stage-specific expression patterns [110], which make them potential biomarkers for specific diseases. Several hundred circRNAs were markedly changed during aging in the human ovary [16], and differential expression of circRNAs has been reported between PCOS patients and non-PCOS individuals [6]. Finally, a large number of circRNAs are enriched in bodily fluids and specifically detected in free-floating cells and extracellular vesicles circulating inside bodily fluids [111,112]. Similar to other ncRNAs, the quantification of circRNAs is mainly determined by RT-PCR using divergent primers. Therefore, circRNAs as biomarkers can be effectively detected in bodily fluid, circulating cells, and extracellular vesicles. To summarize, circRNAs satisfy the primary conditions for consideration as disease biomarkers. In the following sections, recent developments in using circRNAs as potential biomarkers for PCOS are reviewed.

### 5.1. Circular RNAs as Diagnostic Biomarkers in PCOS

The area under the curve (AUC) in receiver operating curve analysis is the main evaluative criterion of diagnostic value. Several circRNAs have been reported to be potential diagnostic biomarkers for PCOS, and their dysregulation is associated with pathological characteristics of PCOS patients. Among them, hsa_circ_0097636 was found to be downregulated in CCs and had good diagnostic efficiency, with an AUC value of 0.738, in a samples of 25 PCOS patients and 25 normal controls. More importantly, binary logistic regression analysis has shown that the AUC value significantly increased to 0.893 when hsa_circ_0097636 was combined with serum testosterone level. Furthermore, hsa_circ_0043533 and hsa_circ_0043532 also achieved high AUC values of 0.709 and 0.718, respectively [49]. Huang et al. verified that three circRNAs (hsa_circ_0085997, hsa_circ_0075692, and hsa_circ_0075691) were dysregulated in PCOS and showed significant distinguishing efficiency with AUC values from 0.75 to 0.89 [104]. These three circRNAs have not been previously reported in other diseases, implying that their specificity and effectiveness make them potential biomarkers for PCOS. However, the sample size of PCOS patients is relatively small in the above studies, and thus a large cohort should be conducted to verify the efficacy of these circRNAs as diagnostic markers for PCOS. Notably, several circRNAs are dysregulated in serum or serum-derived exosomes and plasma, which allows them to be used as potential noninvasive biomarkers for early diagnosis. Therefore, future studies can focus on the alteration of circRNAs in body fluids to monitor PCOS progression.

### 5.2. Therapeutic Role of Circular RNAs in PCOS

The main therapeutic strategies for PCOS patients aim to alleviate clinical symptoms and improve prognostic outcomes. These strategies include medical approaches, surgical procedures, lifestyle modifications, cognitive behavioral therapy, and microbiota-targeted interventions [113,114,115]. Current pharmacological approaches mainly focus on fertility treatment, such as oral contraceptives to regulate the menstrual cycle, letrozole and clomiphene citrate to induce ovulation, inositols to improve metabolic and endocrine aspects, and antiandrogen therapies for the inhibition of high androgen-related symptoms [116]. Surgical interventions have been proposed as effective strategies for PCOS. Laparoscopic ovarian drilling can effectively promote ovulation rate when patients do not respond to medicine [117]. Bariatric surgery is an option for severely obese patients to lose weight and alleviate PCOS symptoms [118]. Lifestyle interventions involve adopting healthy diets, engaging in physical activity, reducing sedentary behaviors, and implementing behavioral strategies to achieve weight loss. Weight loss contributes to decreased testosterone concentration, increased insulin sensitivity, and normalized ovarian function [119]. A randomized controlled trial reported that a three-component treatment, consisting of diet, exercise, and cognitive behavioral therapy, effectively improved emotional wellbeing in obese women with PCOS [120]. The study showed that cognitive behavioral therapy may be a kind of ideal auxiliary treatment method for PCOS. Emerging evidence indicates that intestinal microbiota affects the pathogenesis and progression of PCOS by modulating hormone secretion, gut–brain mediators, cytokines, and metabolite production [121]. This implies that precise regulation of the intestinal microbiome may be a potential therapy for PCOS. For this purpose, probiotic, prebiotic, and synbiotic supplementation has recently been tried as a new treatment for PCOS. The results showed that microbiota-targeted interventions have favorable effects on the metabolic profile and hormonal profile in women with PCOS [122,123]. However, there is still an urgent need for preventative and targeted treatments for PCOS.

In recent years, circRNAs have also attracted great attention as potential therapeutic targets of PCOS. The increasing number of circRNAs associated with the occurrence and progression of PCOS suggests their potential as therapeutic targets. CircRNAs are typically inhibited using RNA interference-based strategies and overexpressed using expression plasmids [124]. For example, circ_0043532 could serve as a therapeutic target through the miR-182/SGK3 axis, and its silencing was found to suppress GC and KGN cell proliferation and cell cycle process in PCOS [57]. Additionally, circ-FURIN, which is involved in a regulatory axis with the miR-195-5p/BCL2 axis, has been shown to suppress cell proliferation and induce apoptosis of GCs in PCOS with small interfering RNA [58]. Another study indicated that depletion of circ_0030018 could delay the progression of PCOS through miR-136-meditated MIEN1 [24]. Therefore, prohibiting circRNA transcription may alleviate symptoms of PCOS. Overexpression of circPSMC3, sponging miR-296-3p to regulate PTEN expression, could relieve symptoms of PCOS in mice. In vitro functional experiments further validated that circPSMC3 could inhibit proliferation and promote apoptosis via cell cycle progression blockage in KGN cells [105], implying that their exogenous introduction may exert a therapeutic function as vectors. IR is also an important therapeutic target of PCOS; enhancing insulin sensitivity can subsequently reduce IR, facilitate glucose metabolism, lower androgen levels, and augment fertility [125]. In a previous study, silencing circANKRD36 inhibited IR by targeting miR-145 via XBP1 [126]. CircRNF111 protects against IR and lipid deposition via regulation of the miR-143-3p/IGF2R axis [127]. Therefore, circRNAs might be potential therapeutic targets for IR in PCOS; however, further scientific research is still needed to fully understand their potential and mechanisms of action.

## 6. Conclusions and Future Directions

CircRNAs have attracted great attention in the field of ncRNAs and have been found in different diseases [128,129], but the study of circRNAs in PCOS is still in its infancy. We comprehensively summarized the biological function and clinical value of circRNAs in PCOS.

Recent studies have shown that a large number of circRNAs are aberrantly expressed in PCOS patients compared with normal controls, some of which have established biological roles or clinical significance. These biological roles are involved in the initiation and progression of PCOS, such as cell proliferation, apoptosis, and steroidogenesis [6]. While circRNAs are a novel frontier in PCOS research, only a few functional roles or clinical applications of circRNAs have been established. A piece of accumulative evidence has shown that circRNAs play a vital role in signaling pathways implicated in metabolic diseases, including glucose and lipid homeostasis. Dysregulation of circRNAs modulate insulin secretion, β-cell function, and insulin signaling in target tissues [12]. Additionally, circRNAs have been verified that participate in lipid metabolism regulation and the development of lipid disorder disease [81]. The results of these studies implied that dysregulation of circRNAs may be associated with the metabolic consequences of PCOS. Therefore, exploring the metabolic functions of circRNAs in PCOS should not be ignored. Further studies on the metabolic regulation of circRNAs will help reveal the mechanism of circRNAs in metabolic disorders associated with PCOS.

Previous studies have mainly focused on the ceRNA hypothesis in PCOS, wherein circRNAs regulate gene expression by acting as a sponge for miRNAs. Current studies show that circRNAs can exert their function via encoding protein- and RNA-based regulatory mechanisms in various physiological and pathological processes [130,131]. For example, circZNF609 can be translated into a protein in myogenesis [132], and it also can sponge miR-483-3p to promote gastric cancer progression [133]. Similarly, circ-SHPRH sponges miR-331-3p and miR-338-5p to inhibit cell proliferation and migration in non-small cell lung cancer [134], and it can generate a peptide SHPRH-146aa to suppress glioma tumorigenesis [135]. However, the role of circRNA translation in PCOS has received little attention, which is in the way of their clinical applications. Therefore, it is necessary to further explore the precise mechanism of circRNA translation in PCOS. Efficient use of circRNA bioinformatics tools, such as IRESite [136], CPC [137], and PhyloCSF [138], may provide an ideal way to identify circRNA coding potential, that is, to investigate circRNA coding potential and further explore the relationship between these peptides and PCOS-related phenotypes. Furthermore, previous studies have found that the progression of PCOS is mainly regulated by conventional factors such as CYP19, IRS, SHBG, and INS [139]. CircRNA databases and software like KEGG or circBase can help find circRNAs’ potential target miRNAs or proteins and further investigate the relationship between these target molecules and conventional factors involved in the pathogenesis of PCOS.

Currently, circRNAs are considered potentially novel diagnostic and prognostic biomarkers in various diseases, including human reproductive diseases [11]. Compared to conventional biomarkers, circRNAs have higher sensitivity and specificity in diagnosis and prognosis, and they play an increasingly important role in various pathophysiological events [106]. Previous studies have revealed that a few circRNAs have the potential to be biomarkers in PCOS. Disease-oriented critical circRNA screening technology is required for further improvement, which can simultaneously analyze differentially expressed circRNAs in different tissues and screen out key circRNAs, providing valuable biomarkers for the diagnosis or therapy of PCOS. Furthermore, recent studies have revealed that some circRNAs can serve as critical metabolic regulators [12], and these findings may provide attractive therapeutic targets for metabolic consequences in PCOS. Moreover, circRNAs combined with traditional biomarkers may exhibit higher diagnostic or prognostic accuracy than single traditional biomarkers. Future research can focus on applying a combination of circRNAs and other biomarkers in diagnosing and treating PCOS patients.

Although it is suggested that circRNAs exert crucial effects in the progression of PCOS, many open questions ought to be considered. First, the sample quantities of previous studies are relatively small. Thus, larger subject cohorts and patients at different stages should be recruited to validate circRNAs biological function and precise mechanisms in PCOS. Second, the detection of some circRNAs needs patient tissue for diagnosis, which causes physical trauma for patients with PCOS. Noninvasive and accessible methods of circRNA detection should be explored in PCOS. Moreover, some circRNAs with low expression are difficult to detect and recognize in body fluids. Detection methods must be improved to enable increased precision in the detection of free circRNAs. Nevertheless, the development of related approaches, such as single-cell RNA sequencing, spatial transcriptomic, and multiomics approaches, are expected to provide valuable insights into the precise mechanisms of circRNAs in PCOS and facilitate their use in the prevention, diagnosis, and treatment of this disorder.

## Figures and Tables

**Table 1:** Dysregulated circRNAs in different reproductive cells or tissues of PCOS patients.

Tissue/Cell Type	Clinical Sample (No. of PCOS and Control)	Dysregulated circRNAs	Validated circRNAs	Enriched Pathways	Reference
Fetal side of placental	3 PCOS vs. 3 control	115 differentially expressed circRNAs (4 downregulated and 101 upregulated)	Downregulated: circ_0023942, circ_0002151, circ_0001274 and circ_0008514		[46]
Granulosa cells	15 PCOS vs. 15 control	27 differentially expressed circRNAs (23 downregulated and 4 upregulated)	Upregulated: hsa_circ_0001577 Downregulated: hsa_circ_0020093	Chemokines, PI3K–Akt and vascular endothelial growth factor signaling pathways	[47]
	6 PCOS vs. 6 control	575 differentially expressed circRNAs (362 upregulated and 213 downregulated)	Upregulated: hsa_circ_0083952, hsa_ circ_0082709, hsa_circ_0002425 and hsa_circ_0015168		[48]
Cumulus cells	6 PCOS vs. 6 control	286 differentially circRNAs (167 upregulated and 119 downregulated)	Upregulated: hsa_circ_0043533 and hsa_circ_0043532 Downregulated: hsa_circ_0097636	Cell cycle, oocyte meiosis, progesterone-mediated oocyte maturation, the FOXO signaling pathway, phosphatidylinositol signaling and glycerophospholipid metabolism	[49]
	20 PCOS vs. 20 control	1032 differentially circRNAs (311 upregulated and 721 downregulated)	Downregulated: hsa_circ_0083952, hsa_circ_0082709, hsa_circ_0002425 and hsa_circ_0015168	Metabolic pathways	[21]
Follicular fluid	5 PCOS vs. 5 control	16 differentially expressed circRNAs (5 upregulated and 11 downregulated)	Upregulated: circRNA (chrM:2262-2426-) and circRNAs (chr13:110076504-110076682+) Downregulated: hsa_circ_0005069, circLDLR and circRNA (chr15:68352856-68353111+)	Ovarian steroidogenesis, aldosterone synthesis and secretion, thyroid hormone signaling pathway, ubiquitin mediated proteolysis, Jak-STAT signaling pathway and endocytosis	[50]
	3 PCOS vs. 3 control	412 differentially expressed circRNAs (167 upregulated and 245 downregulated)	Upregulated: ciRNA-7323_TIAM1, circRNA- 6335_MAN1A2, circRNA-15112_UBAP2, circRNA-9160_CLEC16A, circRNA-14867_ASH1L, circRNA-12884_BPTF, ciRNA-1880_RHOA, ciRNA-10775_RUVBL1 and ciRNA-2003_BECN1	Bacterial infection, associated chronic inflammation, and oxidative stress	[20]

**Table 2:** Circular RNA as a potential biomarker in PCOS.

Biomarker Type	CircRNA	Tissue/Cell Type	Expression Pattern	Proposed Functions or Roles	Reference
Diagnostic biomarker	hsa_circ_0043533	Cumulus cells	Increase	AUC value of 0.709	[49]
	hsa_circ_0043532		Increase	AUC value of 0.718	
	hsa_circ_0097636		Decrease	AUC value of 0.738 (hsa_circ_0097636 alone) AUC value of 0.893 (hsa_circ_0097636 in combination with serum testosterone)	
	hsa_circ_0075691	Follicular fluid	Increase	AUC value of 0.89	[104]
	hsa_circ_0075692		Increase	AUC value of 0.80	
	hsa_circ_0085997		Decrease	AUC value of 0.75	
Therapeutic biomarker	circ_0043532	Granulosa cells	Increase	Silencing circ_0043532 suppressed granulosa cell proliferation and induced apoptosis in PCOS through miR-182/SGK3 axis	[57]
	circ-FURIN	Granulosa cells	Increase	Knockdown of circ-FURIN suppressed proliferation and induced apoptosis of granular cells in PCOS via miR-195-5p/BCL2 axis	[58]
	circ_0030018	Granulosa cells	Increase	Knockdown of circ_0030018 suppressed proliferation, migration, and invasion of KGN cells, while promoting their apoptosis by targeting miR-136/ MIEN1 axis	[24]
	circ_0043533	Granulosa cells	Increase	Knockdown of circ_0043533 inhibited cell viability and proliferation and promoted apoptosis of insulin-treated COV434 and KGN cells by targeting miR-1179	[23]
	circPSMC3	Ovarian tissue	Decrease	CircPSMC3 inhibited cell proliferation and promoted apoptosis by blocking the cell cycle in human-like granular tumor cell lines	[105]

Abbreviations: AUC—area under the curve.

## Data Availability

Not applicable.

## Abbreviations

PCOS	polycystic ovary syndrome
CircRNA	circular RNAs
IR	insulin resistance
ncRNAs	noncoding RNAs
Pol II	RNA polymerase II
EcRNAs	exonic circRNAs
ciRNAs	intronic circRNAs
EIciRNAs	exon–intron circRNAs
ceRNAs	competitive endogenous RNAs
RBPs	RNA-binding proteins
IRES	internal ribosome entry site
m6A	N6-methyladenosines
eIF3	eukaryotic initiation factor 3
RT-PCR	reverse-transcription polymerase chain reaction
GO	Gene Ontology
KEGG	Kyoto Encyclopedia of Genes and Genomes
GCs	granulosa cells
CCs	cumulus cells
FF	follicular fluid
LH	luteinizing hormone
AR	androgen receptor
LDLR	low-density lipoprotein receptor
AMH	anti-Mullerian hormone
TCs	theca cells
LPL	lipoprotein lipase
AUC	area under the curve
